# Representative Percentile Curves of Physical Fitness From Early Childhood to Early Adulthood: The MoMo Study

**DOI:** 10.3389/fpubh.2020.00458

**Published:** 2020-09-11

**Authors:** Claudia Niessner, Till Utesch, Doris Oriwol, Anke Hanssen-Doose, Steffen C. E. Schmidt, Alexander Woll, Klaus Bös, Annette Worth

**Affiliations:** ^1^Institute of Sport and Sport Science, Karlsruhe Institute of Technology, Karlsruhe, Germany; ^2^Department of Pedagogical Assessment and Potential Development, Institute of Educational Sciences, University of Münster, Münster, Germany; ^3^Institute of Movement and Sport, University of Education, Karlsruhe, Germany

**Keywords:** health-related, performance, skill-related, LMS, reference, monitoring, Germany, youth

## Abstract

**Introduction:** Monitoring of physical fitness in youth is important because physical fitness is a summative indicator of health. From a developmental and preventive perspective, physical fitness levels are relatively stable from childhood to early adulthood. Thus, it is important to monitor physical fitness on a population based level being able to intervene at early stages (1). In order to reliably assess and evaluate the physical fitness of youth, a reliable system of standard values based on representative data is required. The aim of this analysis is to report sex- and age-specific physical fitness percentile curves from childhood to early adulthood in a nationwide sample in Germany.

**Methods:** We use data from the nationwide representative Motorik Modul (MoMo) Study in Germany (data collection wave 1: 2009–2012; age: 4–23 years; *n* = 3,742; 50.1% female). Physical fitness was assessed by means of the MoMo test profile covering four dimensions of physical fitness (strength, endurance, coordination, and flexibility) and including eight physical fitness items. Percentile curves were fitted using the LMS transformation method of Cole and Green.

**Results:** Standardized age- and sex-specific physical fitness percentiles were calculated for eight items: ergometric endurance testing, standing long jump, push-ups, sit-ups, jumping side-ways, balancing backwards, static stand, and stand and reach test. The physical fitness curves differ according to gender and the fitness dimension. Physical fitness improvements with age are linear (e.g., max. strength) or curvilinear (e.g., coordination) and have their stagnation points at different times over the course of adolescence.

**Discussion:** Our results provide for the first time sex- and age-specific physical fitness percentile curves for Germany from 4 to 17 years. Differences in curve-shapes indicating a timed and capacity-specific physical fitness development. Nationwide German physical fitness percentiles can be useful in comparing different populations (e.g., cross-country), reporting secular trends, comparing special groups, and to evaluate physical fitness interventions.

## Introduction

Physical fitness levels play a major role in overall healthy child and youth development (2). Representing powerful biomarkers of health status already in early childhood, especially the maintenance of satisfactory fitness levels is highly connected with current public health issues as physical development (3), the prevention of diabetes, obesity (4), cardiovascular disease risk factors (5), cancer and mental health (2, 6). Therefore, it is important to monitor population fitness levels longitudinally to being able to intervene at an early stage (7, 8). The importance of such monitoring has also been shown by critical decreases in physical fitness levels in a large world-wide sample (9).

The specific selection of appropriate definitions, assessments and subsequent test interpretations are important in the scientific as well as practical fields of human health and sports sciences. The construct of physical fitness consists of two components (10): on the one hand, health-related fitness includes cardiovascular endurance, strength endurance, explosive muscular strength, body composition as well as flexibility (10). On the other hand, skill-related fitness is defined as agility, balance, coordination, speed, power and reaction time (10). Physical fitness can be accessed via laboratory as well as field tests. Although laboratory tests using sophisticated material are capable of providing very objective and detailed outcomes, these tests are not suitable for comprehensive monitoring of physical fitness levels in large scale studies across cities, states or countries. Here, single field tests assessing specific fitness domains are often utilized and gathered in standardized test batteries to comprehensively cover physical fitness [e.g., FitnessGram (11), Eurofit (12), German Motor Performance Test 6–18 (13)]. Among various widely known and broadly used single test items, the MoMo Motor Performance Test (14) was compiled using the most widely used single item tests in Europe in order to broadly and longitudinally assess physical fitness from early childhood to early adulthood.

In Germany, it has been shown that certain physical fitness components are relatively stable between cohorts from childhood to early adulthood while others increase or decrease (3, 15). In order to successfully monitor physical fitness levels over time in a representative manner, it is necessary to provide up-to-date normative data of the specific test items. Many researchers, but also physical education teachers or sport coaches assess physical fitness from their students and need valid and simple possibilities judging their physical fitness performance (16). Therefore, this study aims to provide up-to-date age- and sex-related percentile curves of persons aged four to 17 years for frequently used test items:

(1) Ergometric endurance testing, (2) standing long jump, (3) push-ups, (4) sit-ups, (5) jumping side-ways, (6) balancing backwards (7) static stand, and (8) stand and reach test.

## Methods

The study was conducted according to the Declaration of Helsinki. Ethics approval was obtained by the University of Konstanz (Wave 1). The Federal Commissioner for the “data protection” and “freedom of information” was informed about the study and approved it.

### Participants

Data were obtained from the nationwide German Motorik-Modul study (MoMo). The MoMo study is an in-depth module study of the German Health Interview and Examination Survey for Children and Adolescents which was conducted by The Robert Koch-Institute (RKI, Berlin) since 2003 (17, 18). The MoMo study provides nationwide representative data on the physical fitness and physical activity status (19). The study was set up in 2003 (2003–2006 MoMo Baseline). Two consecutive survey waves were conducted until now: MoMo Wave 1 (2009–2012) and MoMo Wave 2 (2014–2017). Children and adolescents were invited to the physical fitness tests at central locations within close proximity to their homes in the 167 cities and municipalities.

The data of 3,284 children and adolescents (1,644 female, 1,646 male) aged 4–17 years were reported in figures below while LMS curve modeling used data of persons till 23 years (*N* = 3,742; male 49.9% *N* = 1,868; female 50.1% *N* = 1,874) from the MoMo Wave 1 survey (2009–2012) was used to model physical fitness percentiles of children and adolescents. We have modeled the percentile curves up to 23 years, as the model fit for the percentile curves become better and more accurate with more data and thus over a larger age range. From the age of 17 years on, however, our sample is no longer representative for Germany, so we report only the data up to 17 years. Characteristics of the whole sample including individual level of socioeconomic status (SES) (20), BMI (21), and type of residential area (22) differentiated by age group and sex are shown in Supplement Table 1 in the Supplement Material.

Detail information about mean values of all physical fitness tests for age groups and gender can be found here (23).

### Sampling

To ensure a diverse sample of German children and adolescents, a nationwide, stratified, multi-stage sample with two evaluation levels was drawn (24).

First, a systematic sample of 167 primary sampling units was selected from an inventory of German communities that were stratified according to the BIK classification system that measures the level of urbanization and the geographic distribution (17). The probability of any community being picked was proportional to the number of inhabitants younger than 18 years in that community. Second, an age stratified sample of randomly selected children and adolescents was drawn from the official registers of local residents. At the second measurement point (KiGGS Wave 1 study), 12,368 children and adolescents participated (18). This sample built the population for the MoMo Wave 1 subsample. 6,076 from KiGGS Wave 1 were randomly assigned to MoMo Wave 1. From those, 3,994 participated in MoMo (65.7%). After excluding participants without a valid physical fitness test (one test item was sufficient), a total of 3,742 children and adolescents 4–23 years remained for this analysis.

### Representativeness

Weighting procedure was used to account for potential bias in outcome variables caused by selective unit nonresponse (24). In the first step, inverse probability weights were applied via logistic regression to eliminate differences in outcome variables between the MoMo subsample and the weighted representative KiGGS sample. In the second step, the MoMo subsample was stratified using data of the German Micro Census 2010 to ensure representativeness of the target population (German children and adolescents aged 4–17 years) regarding sex, age, region, migration background, and education level (25).

### Material

The MoMo Motor Performance Test (14) was developed in order to broadly cover the construct of physical fitness. The physical fitness tests were carried out in the time frame from 8 a.m. to 6 p.m. Trained testing stuff conducted the tests with the children and adolescents in a one-to-one supervision. Testing always start with the coordination tasks followed by the strength tasks and at the end the endurance test on the bike ergometer was carried out. The duration of the test is about 60 min. The test items originated from common validated test profiles and were pretested, optimized, discussed with experts, and documented in a comprehensive test manual (14). The overall reliability was calculated using the standardized total value and results in a correlation of *r* = 0.97 (*p* = 0.00) and no significant difference in mean value. The objectivity (tested using different test directors) is very good (*r* = 0.98 to 0.99), the percentage difference is less than one percentage point for all test items (26). It consists of eight items covering health-related (i.e., endurance, strength endurance, lower body explosive muscular strength, and flexibility) and skill-related fitness domains (i.e., balance, coordination), which are covered by the following test items:

*Cardiovascular endurance* was assessed using a static bicycle ergometer test. It measures the aerobic endurance capacity of participants using a sequential step test design. The test is started at a calculated input load of 0.5 watt/kg body weight and a cadence of 70 revolutions per minute (rpm). Each load level is held for two minutes. Then the load is increased by 0.5 watts per kilogram of body weight. Each level is shown on a digital display. For the assessment of the performance the power at a heart rate of 170 bpm (Physical Working Capacity [PWC170]) were used. The test stops at three occasions: (1) if there is a pulse of above 190 (participants up to 10 years) or above 180 (participants from 11 years), respectively, (2) if the cadence falls below 50 rpm for more than 20 s, or (3) if participants want to stop due to subjective exhaustion. Children aged 4–5 years did not participate in the test.

*Strength endurance* is assessed for upper extremities as well as body core via sit-ups and push-ups. Firstly, participants perform sit-ups to cover body core strength endurance in a lying position with bent legs. The test instructor fixes the feet on the ground. The fingertips touch the temples in order to avoid pulling the neck. At each sit-up, participants need to touch the knees with their elbows without lifting the basin. Subjects repeat as many correct sit-ups as possible in 40 s. Secondly, push-ups were performed in order to cover upper extremities strength endurance starting with hands together at the lower back. In a first step, participants push their body up with a plank body angle. When the arms are straight, subjects touch one hand with the other hand and return the starting position with constant body control. The resulting variable is the number of repeated correct push-ups in 40 s. Children aged 4–5 years did not partake in these two tests.

*Explosive muscular strength* of the lower extremities is assessed via standing long jump. Participants start at a line, which is marked on a tartan mat. It is important that subjects jump using both legs together and do not fall backwards after landing the jump. Otherwise, the jump has to be repeated. Two jumps were performed while the maximum jump is counted.

*Coordination including Balance* is assessed via jumping sideways, static stand and balancing backwards. For the jumping sideways task the test instructor creates two adjacent 50 cm squares with 5 cm lines. Participants have to jump from one to the other square like a pendulum jumping with both legs without touching the boundaries. After a 1-min pause, subjects have a second trial. The test instructor counts the number of correct jumps in 15 s.

*Balance* is assessed via static stand and more dynamic balancing backwards. Firstly, static jump measures sensomotoric regulation of precision tasks on a 3 cm broad beam. Participants have to balance on the beam without touching the own leg, the beam or the ground. Test instructor counts the number of mistakes in one minute. Secondly, balancing backwards assesses dynamic whole-body balance on three differently broad. Subjects have two trials each on a 6 cm, on a 4.5 cm, and on a 3 cm beam. Test instructor counts the number of steps from each participant before touching the ground while reaching a maximum of eight steps per beam (total max. 48 steps).

*Flexibility* of the lower extremities is assessed via stand and reach. Stand and reach specifically measures the flexibility of the lower back and hamstring muscles. Test instructors measure the level of the fingers with the feet recording to zero. Negative values are associated with not being able to reach one's own toes while positive values are related to reaching further than the toes. Participants have to stay in the final position for two seconds in order to avoid swinging execution of the task. Subjects have two trials for this test.

### Data Analysis: LMS Curves

Age- and sex-specific percentile values (P5, P10, P15, P30, P50, P70, P85, P90, and P95) were calculated and percentile curves were fitted using the LMS transformation method of Cole and Green (27). The LMS method summarizes the changing distribution by three curves representing the median (M), coefficient of variation (L), and skewness (S) (expressed as a Box-Cox power). Using penalized likelihood the three curves can be fitted as cubic splines by non-linear regression, and the extent of smoothing required can be expressed in terms of smoothing parameters or equivalent degrees of freedom (27). The values of L, M and S were constrained to change smoothly with age, and the fitted values can be used to construct any required percentile curves (28). All analyses were performed with LMS chartmaker pro (V. 2.3). When modeling some extreme values resulted in the sample of computational instabilities in the application of the method. For this reason, values were excluded which exceeded plus and minus three standard deviations. The number of degrees of freedom for the splines is considered optimal, following a recommendation by Cole (29), if the model's deviance (P Deviance and SBC Black Bayesian Criterion) with a further increase of 1° of freedom does not improve by more than 8. In addition, a visual quality control was conducted: Empirical and fitted centiles plotted on top of each other. This is an accurate technique in which the observations are divided into age groups (half-yearly). Empirical centiles are computed for each group, and these are plotted together with the fitted curves. If everything is right, the fitted curves should be close to the point estimates (that is, within sampling error). Quantile–quantile plot (Q–Q plot) of the *z*-scores were also applied. The display plots the quantiles of the theoretical distribution (on the horizontal axis) against those of the empirical distribution (on the vertical axis).

## Results

**Figure 1:**
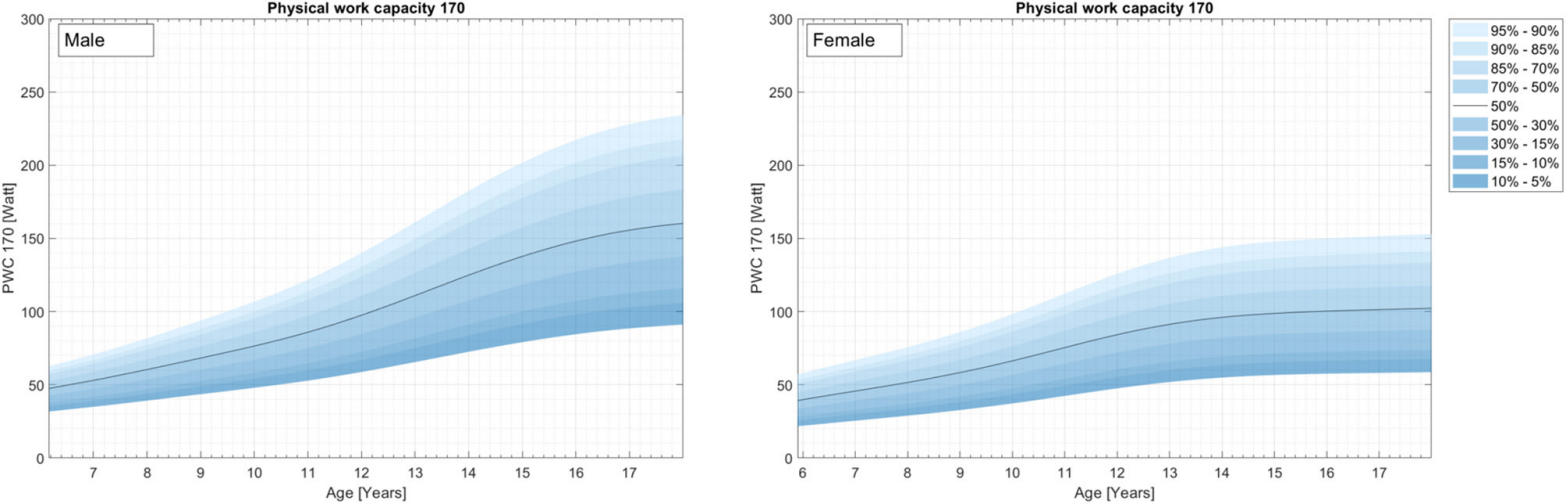
Cardiovascular endurance curves (Physical Work Capacity 170, ergometric bicycle test) for the representative sample of German boys and girls aged 4–17 years.

**Figure 2:**
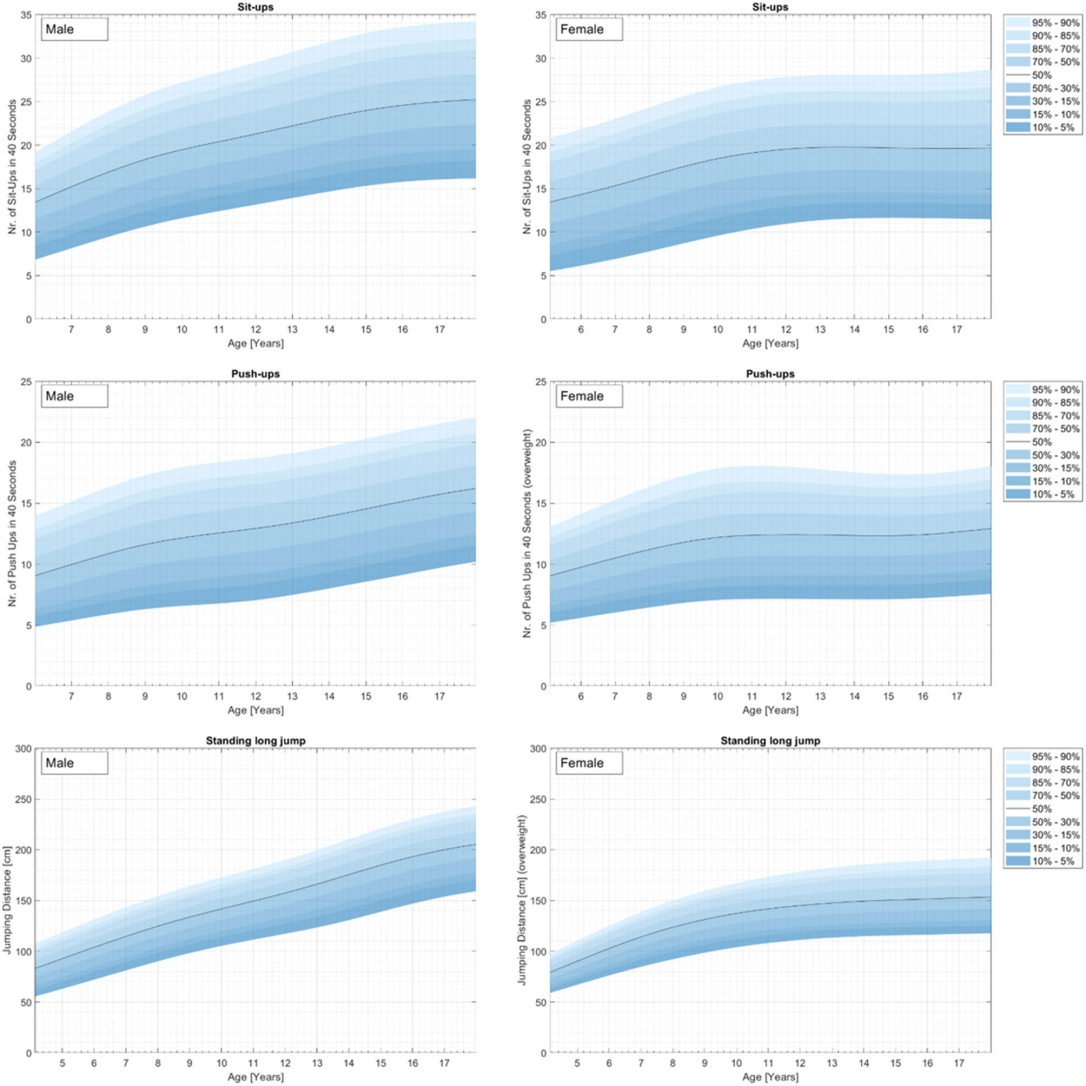
Strength curves for the representative sample of German boys and girls aged 4–17 years for the testitems Sit-ups (top), Push-ups (middle), and Standing long jump (bottom).

**Figure 3:**
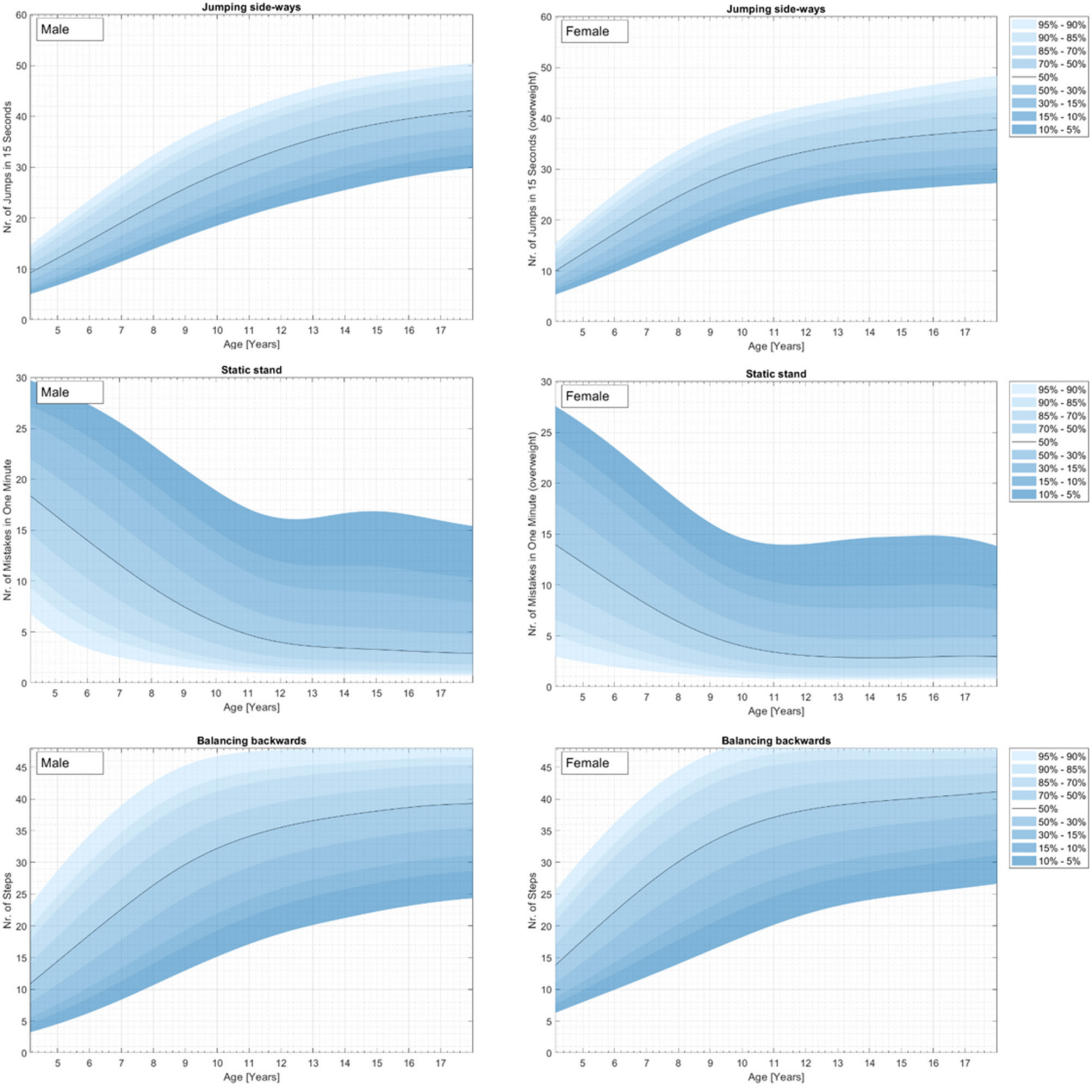
Coordination curves for the representative sample of German boys and girls aged 4–23 years for the test items jumping side-ways (top), static stand (middle), and balancing backwards (bottom).

**Figure 4:**
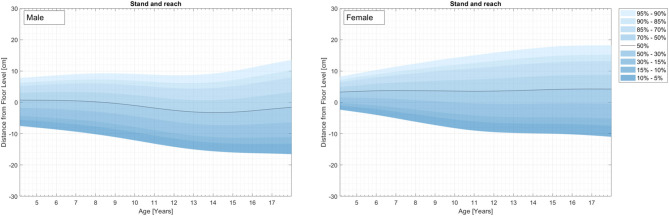
Flexibility curves (Stand and reach) for the representative sample of German boys and girls aged 4–23 years.

Physical fitness indicators for 3,742 children and adolescents from a representative (for 2010) nationwide German sample were used to develop percentile curves for male (49.9%) and female (50.1%) students aged 4–23 years. Supplement Table 1 and Figures 1–4 demonstrate the distributions and corresponding standardized age-based percentiles of all items separated for male and female subjects. We present figures to reproduce the shape of the percentile curves but at the same time provide the data basis for concrete physical fitness comparisons using the data in the tables for practical use.

### Cardiovascular Endurance

The results for cardiovascular endurance can be found in Figure 1 and Supplement Table 2.

Although the lowest percentile curves for fitness are relatively flat, a steady increase in mean aerobic performance is seen as children enter adolescence. On average, girls had a maximum fitness between ages 14 and 15 years, while boys, on average, had a maximum fitness between 15 and 16 years. Both sexes demonstrated a slight decrease after these peaks.

### Strength

#### Sit-Ups

Results for strength are represented in Figure 2 and Supplement Tables 3–5.

There is a steady increase in mean strength performance from childhood to adolescence. On average, girls had a maximum fitness between ages 11 and 12 years, while boys, on average, reach their maximum performance probably at the 17–18 years. The girls demonstrated a slight decrease after the peak.

#### Push-Ups

For females, the lowest percentile curves for fitness are relatively flat. On average, girls had a maximum strength performance at the age of 11 years (3). Boys also demonstrate a steady increase in mean strength performance over childhood and adolescence for all percentile curves except for the highest percentiles. The female percentile curve demonstrates a slight decrease after the peak for the highest percentiles or a stabilization for the other percentile curves.

#### Standing Long Jump

There is a steady increase in mean strength performance of the standing long jump from childhood to adolescence for both sexes. On average, standing long jump performance in girls stabilizes at the age of 11 years, while boys, demonstrate a steady increase. Both sexes demonstrated no decrease in this timespan.

#### Coordination Including Balance

Figure 3 and Supplement Tables 6–8 show the results for coordination including jumping sideways, static stand, and balancing backwards.

#### Jumping Side-Ways

There is a steady increase in mean jumping sideways performance from childhood to adolescence for both sexes. On average, performance stabilizes for both sexes at the age between 12 and 14 years. Both sexes demonstrated no decrease in this timespan.

#### Static Stand

There is a steady decrease in mean ground contact from childhood to adolescence for both sexes up to 10–12 years. After that, performance stabilizes. There is a ceiling effect for the high percentiles.

#### Balancing Backwards

There is a steady increase in mean balancing performance from childhood to adolescence for both sexes. On average, performance stabilizes for both sexes at an age of 9 years. Both sexes demonstrated no decrease in this timespan.

#### Flexibility

Results for flexibility are represented in Figure 4 and Supplement Table 9.

#### Stand and Reach

In mean, both sexes demonstrate relatively flat percentile curves. Percentiles show a siccors effect with increasing age between the low and the high percentile curves. The low percentile curves decrease steady whereas high percentile curves increase steady from childhood to adolescence.

## Discussion

Poor physical fitness levels are associated with negative health trajectories from early childhood to adulthood e.g., (2, 4, 6). Therefore, it is an important public health goal to improve and maintain physical (i.e., skill-related and especially health-related) fitness levels of children and adolescents as well as their physical self-concepts in order to foster physical activity behavior, e.g., (30, 31). In this regard, public health as well as politics need reliable and valid assessments and representative normative data in order to validly classify people's current fitness level according to their sex and current age. Unfortunately, these normative data did not exist for all fitness assessments and representative samples so far. Therefore, the aim of this study was to provide normative LMS percentile curves for several physical fitness parameters for typical developed German children and adolescents. Using LMS curves provides several advantages for representative normative data. Firstly, the sample size of each age group can be relatively small as the age and sex dependent groups were in the current study, because each age group benefits from adjacent age groups which increases overall accuracy and power of the normative data. Secondly, these adjacent age groups increase the developmental character of the normative data for longitudinal analyses purposes as it has been established for BMI (32) and Bioelectrical Impedance percentiles (33). This is clearly a benefit but also a limitation of this study, because smoothing algorithms might damage the validity of the data under specific circumstances. Therefore, the LMS curves were examined in-depth theory-driven.

Fitting MLS percentile curves for Germany (MoMo Wave 1, 2009–2012) enables us to compare the physical fitness in Germany with other nations which have percentile curves and using same test items. As an example for using our percentile curves we conducted a comparison for the test item standing long jump, as a highly standardized test item, with current European standard values of Tomkinson et al. (34). Boys (9–17 years) in Germany perform worse than their European peers in almost all age groups (e.g., boys 17 year. German P50 = 200,00 cm, European P50 = 205.8 cm). The standing long jump performance of German girls (9–17), on the other hand, is comparable to the European peer group (1). There are some other countries presenting physical fitness percentile curves [Australia: (35), Spain: (36), US Wisconsin: (37), Europe: (34)]. These percentile curves partly allow comparisons between countries, but these comparisons must be interpreted with caution, since the test items, the survey periods, and the samples differ. At first sight, physical fitness levels of populations have a continuous character rather than dichotomous (strict criterion-based “healthy” or “non-healthy”). Hence, it is intuitive to expect most information about children within very detailed percentiles and such population-based percentile distribution curves (and values) can be seen as very useful for public health cross-sectional assessment and prospective evaluation of interventions. However, from a test theoretical point of view (i.e., considering measurement error, test instructor effects, etc.), motor test performance should be interpreted in validated categorization system (or in percentile ranges) instead of single percentile values, because development is rarely truly (38–41). In this regard, percentile curves were provided based on internationally comparable values (i.e., percentiles 5, 10, 20, 30, 50, 70, 80, 90, and 95 as well as interim curves) which have successfully been shown for the measurement of physical fitness across childhood (32, 34, 42).

How “high” the level of physical fitness should be has not been sufficiently researched so far, but it is assumed that certain threshold values of physical fitness should be reached by every child to ensure growing up healthy. Some authors give a fitness level of less than percentile 5 as an indicator of health risks and thus define this value as the boundary between “healthy” and “increased risk for diseases” e.g., (33, 43). The problem with this definition might be that it is based on standard values and do not provide information regarding how the values relates to health (44). The only health-related fitness test that tries to circumvent this problem is the FitnessGram test battery (11) and the VO_2_max test (45). However, the test items used at Fitnessgram correspond to the type of test used in the MoMo study (e.g., push-up, sit-up, etc.), but they differ in terms of their execution and/or duration. As a result, the health-related fitness cut-off points defined in the FitnessGram cannot be transferred to the MoMo study. However, the determination of cut-off points and so define thresholds on the basis of the MoMo study is in progress, e.g., we are working on the conversion of the PWC 170 values of our study into VO2max values via ergonizer software for sports medical performance diagnostics. The aim is to use the thresholds identified by Ruiz et al. (45) in their systematic literature search for studies that determined a cardiorespiratory fitness cut point that predicted cardiovascular disease risk in children and adolescents.

In Conclusion, we present LMS coefficient relating to representative physical fitness percentile ranges in order to provide cut-offs for children with positive but also negative fitness values. The new LMS curves are available by year from age 4 to 17 years. These LMS coefficient make it easy to compare children's physical fitness levels within one age branch, but also between age brands and longitudinally. We recommend to use these new, rather than old, LMS curves to transform children's raw performances into standard values that enable the identification of children especially in the peripheral areas (16). Finally, we emphasize that the presented normative data should be regularly updated and put in to context with older data to monitor trends in physical fitness performance.

## Data Availability Statement

The raw data supporting the conclusions of this article will be made available by the authors, without undue reservation.

## Ethics Statement

The studies involving human participants were reviewed and approved by Karlsruhe Institute of Technology. Written informed consent to participate in this study was provided by the participants' legal guardian/next of kin.

## Author Contributions

CN and TU drafted the initial manuscript and reviewed and revised the manuscript. CN and DO coordinated and supervised data collection. CN, DO, and SS modeling the percentile curves. AWol, AWor, and KB conceptualized and designed the study. AH-D supervised data collection and critically reviewed the manuscript for important intellectual content. All authors have read and approved the submitted manuscript.

## Conflict of Interest

The authors declare that the research was conducted in the absence of any commercial or financial relationships that could be construed as a potential conflict of interest.
