# Supplementary material for: Representative Percentile Curves of Physical Fitness From Early Childhood to Early Adulthood: The MoMo Study
Source: Front Public Health. 2020 Sep 11;8:458. doi: 10.3389/fpubh.2020.00458 (PMC7516256; doi:10.3389/fpubh.2020.00458)
Supplement: Supplementary file 1 [file Data_Sheet_1.PDF]

Supplementary Material

1 Supplementary table of description of the analysed subsample

Tab.1. Descriptive statistics of the whole sample used for LMS modeling N=3,742 (MoMo Wave 1 subsample physical fitness, unweighted sample)

	**Male (N=1,868)**					**Female (N=1,874)**				
	**4-5 years**	**6-10 years**	**11-13 years**	**14-17 years**	**18-23 years**	**4-5 years**	**6-10 years**	**11-13 years**	**14-17 years**	**18-23 years**
**N**	165	571	441	463	263	165	594	425	454	263
**Sex %**	49.7	49.0	50.7	50.5	48.9	50.3	51.0	49.3	49.5	51.1
**Age [years]**	5.00 ±0.51	8.40 ±1.48	12.46 ±0.86	15.89 ±1.14	20.54 ±1.69	5.10 ±0.53	8.50 ±1.48	12.47 ±0.85	15.82 ±1.16	20.60 ±1.75
**SES high %**	34,5	29,4	27,0	24,5	41,7	32,9	28,3	23,4	25,1	33,3
**SES low %**	4,8	7,4	8,4	11,0	0,0	5,4	5,5	9,8	8,8	0,0
**TRA: Rural (< 5,000 inhabitants) %**	28,5	26,6	24,9	25,5	20,6	26,3	25,0	27,8	19,8	22,6
**TRA: Metropolitan (100,000 inhabitants +) %**	10,9	14,4	16,1	11,7	14,5	14,4	14,8	12,9	16,7	18,3
**BMI [kg/m^2]**	15.75 ±1.30	16.89 ±2.47	19.47 ±3.65	21.53 ±3.55	22.78 ±4.77	15.56 ±1.33	16.87 ±2.88	19.39 ±3.23	21.68 ±3.62	22.95 ±4.61
**Overweight [%]***	8.5	14.0	15.4	13.2	20.6	6.7	13.7	14.8	13.2	13.7
**Obese [%]***	1.8	3.2	4.8	4.8	7.5	1.2	3.4	2.6	4.0	7.6

BMI measurements: height, weight surveyed by trained staff with stadiometer Seca 213, Scale Seca 813 robusta. BMI was catogrized using IOTF Values of Cole & Lobstein (2012) | SES: socio economic status (Lampert et al. 2014) | TRA: Type of residential area (Hoffmeyer-Zlotnik, 2005)

2 Supplementary tables of physical fitness percentiles

Tab. 2: Cardiovascular endurance Percentiles (Physical Work Capacity 170) for the representative sample of German boys and girls aged 4-17y (MoMo Wave 1)

Boys PWC 170 [Watt]

Age (years)	5%	10%	15%	30%	50%	70%	85%	90%	95%
**6**	31,77	35,33	37,70	42,60	47,54	52,41	57,11	59,35	62,63
**6,5**	33,02	36,77	39,28	44,46	49,70	54,88	59,89	62,27	65,78
**7**	34,96	39,00	41,72	47,34	53,06	58,74	64,25	66,87	70,76
**7,5**	37,02	41,38	44,31	50,41	56,65	62,87	68,93	71,82	76,11
**8**	39,18	43,86	47,03	53,64	60,43	67,24	73,89	77,08	81,81
**8,5**	41,37	46,39	49,79	56,94	64,32	71,74	79,03	82,54	87,75
**9**	43,56	48,92	52,58	60,28	68,27	76,35	84,32	88,15	93,87
**9,5**	45,74	51,47	55,38	63,67	72,30	81,06	89,73	93,92	100,18
**10**	47,96	54,07	58,26	67,16	76,48	85,97	95,40	99,96	106,79
**10,5**	50,31	56,85	61,34	70,92	80,98	91,26	101,51	106,48	113,93
**11**	52,85	59,88	64,71	75,04	85,92	97,08	108,23	113,65	121,77
**11,5**	55,66	63,24	68,46	79,63	91,43	103,56	115,70	121,60	130,47
**12**	58,75	66,94	72,59	84,71	97,52	110,70	123,92	130,36	140,03
**12,5**	62,06	70,92	77,04	90,17	104,07	118,39	132,75	139,75	150,27
**13**	65,52	75,09	81,71	95,91	110,94	126,44	141,98	149,56	160,94
**13,5**	69,05	79,36	86,48	101,77	117,96	134,64	151,38	159,53	171,78
**14**	72,56	83,59	91,22	107,57	124,89	142,72	160,60	169,32	182,41
**14,5**	75,93	87,65	95,75	113,12	131,49	150,40	169,35	178,58	192,43
**15**	79,09	91,46	100,00	118,31	137,65	157,54	177,45	187,14	201,69
**15,5**	81,98	94,94	103,88	123,03	143,23	163,99	184,75	194,85	210,00
**16**	84,53	98,01	107,30	127,18	148,13	169,63	191,12	201,56	217,22
**16,5**	86,70	100,63	110,21	130,70	152,27	174,39	196,45	207,17	223,24
**17**	88,50	102,78	112,60	133,57	155,63	178,21	200,71	211,64	228,01

Girls PWC 170 [Watt]

Age (years)	5%	10%	15%	30%	50%	70%	85%	90%	95%
**6**	22,03	25,92	28,56	34,11	39,85	45,63	51,32	54,05	58,11
**6,5**	23,76	27,89	30,71	36,65	42,81	49,03	55,17	58,12	62,52
**7**	25,46	29,84	32,82	39,14	45,71	52,38	58,96	62,14	66,87
**7,5**	27,15	31,76	34,92	41,61	48,59	55,70	62,73	66,13	71,21
**8**	28,89	33,75	37,08	44,16	51,58	59,14	66,65	70,29	75,73
**8,5**	30,75	35,88	39,41	46,93	54,82	62,89	70,92	74,81	80,64
**9**	32,76	38,19	41,93	49,93	58,35	66,97	75,57	79,75	86,01
**9,5**	34,92	40,69	44,66	53,18	62,18	71,41	80,64	85,13	91,87
**10**	37,26	43,39	47,63	56,72	66,34	76,25	86,17	91,00	98,26
**10,5**	39,77	46,29	50,81	60,51	70,81	81,43	92,10	97,30	105,12

**11**	42,39	49,30	54,09	64,42	75,40	86,77	98,21	103,80	112,20
**11,5**	45,02	52,30	57,36	68,28	79,94	92,03	104,23	110,20	119,20
**12**	47,55	55,16	60,46	71,93	84,20	96,97	109,89	116,22	125,78
**12,5**	49,86	57,75	63,25	75,19	88,01	101,38	114,93	121,59	131,65
**13**	51,88	60,00	65,67	78,00	91,26	105,13	119,23	126,16	136,64
**13,5**	53,57	61,87	67,68	80,31	93,93	108,20	122,73	129,88	140,71
**14**	54,92	63,36	69,26	82,14	96,03	110,60	125,45	132,77	143,86
**14,5**	55,93	64,48	70,46	83,50	97,59	112,38	127,46	134,90	146,17
**15**	56,67	65,29	71,34	84,51	98,74	113,68	128,91	136,42	147,81
**15,5**	57,19	65,89	71,98	85,26	99,60	114,65	129,99	137,55	149,02
**16**	57,57	66,33	72,47	85,84	100,27	115,40	130,83	138,43	149,95
**16,5**	57,86	66,69	72,86	86,31	100,82	116,04	131,54	139,18	150,75
**17**	58,11	66,99	73,20	86,72	101,31	116,61	132,18	139,86	151,48

Tab. 3: Sit-up Percentiles for the representative sample of German boys and girls aged 4-17y (MoMo Wave 1)

Boys Sit-Ups [Number in 40 sec]

**Age (years)**	**5%**	**10%**	**15%**	**30%**	**50%**	**70%**	**85%**	**90%**	**95%**
**6**	6,84	8,42	9,44	11,47	13,44	15,33	17,11	17,94	19,15
**6,5**	7,48	9,10	10,15	12,26	14,32	16,31	18,19	19,07	20,36
**7**	8,15	9,80	10,88	13,06	15,21	17,30	19,28	20,21	21,58
**7,5**	8,81	10,49	11,59	13,84	16,07	18,25	20,32	21,30	22,74
**8**	9,45	11,15	12,28	14,59	16,89	19,14	21,31	22,33	23,83
**8,5**	10,06	11,79	12,93	15,28	17,65	19,98	22,22	23,28	24,84
**9**	10,63	12,37	13,53	15,92	18,34	20,72	23,03	24,13	25,74
**9,5**	11,14	12,89	14,06	16,48	18,94	21,37	23,74	24,86	26,52
**10**	11,60	13,35	14,53	16,97	19,46	21,94	24,35	25,49	27,19
**10,5**	12,01	13,77	14,95	17,42	19,93	22,44	24,89	26,06	27,79
**11**	12,40	14,16	15,35	17,84	20,38	22,92	25,40	26,58	28,34
**11,5**	12,77	14,55	15,74	18,25	20,82	23,39	25,90	27,11	28,90
**12**	13,15	14,93	16,14	18,67	21,27	23,87	26,43	27,65	29,47
**12,5**	13,53	15,33	16,54	19,10	21,73	24,37	26,96	28,21	30,06
**13**	13,91	15,72	16,95	19,54	22,20	24,88	27,51	28,78	30,66
**13,5**	14,29	16,12	17,36	19,98	22,68	25,39	28,07	29,35	31,26
**14**	14,66	16,51	17,77	20,41	23,14	25,90	28,61	29,91	31,85
**14,5**	15,00	16,87	18,14	20,81	23,58	26,36	29,10	30,42	32,39
**15**	15,30	17,19	18,47	21,17	23,96	26,77	29,54	30,87	32,85
**15,5**	15,56	17,47	18,76	21,48	24,29	27,13	29,91	31,25	33,24
**16**	15,77	17,69	19,00	21,74	24,57	27,41	30,21	31,55	33,55
**16,5**	15,93	17,87	19,19	21,95	24,79	27,65	30,45	31,79	33,79
**17**	16,05	18,01	19,34	22,11	24,97	27,83	30,63	31,98	33,97

Girls Sit-ups [Number in 40 sec]

Age (years)	5%	10%	15%	30%	50%	70%	85%	90%	95%
**6**	6,15	8,05	9,29	11,82	14,32	16,76	19,09	20,19	21,81
**6,5**	6,52	8,45	9,71	12,27	14,81	17,28	19,64	20,76	22,39
**7**	6,91	8,87	10,15	12,75	15,33	17,83	20,22	21,35	23,00
**7,5**	7,34	9,33	10,63	13,26	15,87	18,41	20,84	21,98	23,65
**8**	7,78	9,80	11,11	13,79	16,43	19,01	21,46	22,62	24,32
**8,5**	8,24	10,27	11,60	14,31	16,99	19,59	22,08	23,25	24,97
**9**	8,70	10,74	12,08	14,81	17,51	20,15	22,66	23,85	25,59
**9,5**	9,15	11,20	12,54	15,28	18,00	20,65	23,19	24,39	26,15
**10**	9,58	11,62	12,96	15,70	18,43	21,10	23,66	24,87	26,64
**10,5**	9,98	12,00	13,33	16,06	18,80	21,47	24,04	25,26	27,05
**11**	10,34	12,33	13,66	16,37	19,09	21,77	24,35	25,57	27,37
**11,5**	10,67	12,63	13,93	16,62	19,33	22,01	24,59	25,81	27,62
**12**	10,95	12,88	14,16	16,83	19,52	22,19	24,77	26,00	27,81
**12,5**	11,18	13,07	14,34	16,97	19,65	22,31	24,89	26,12	27,94
**13**	11,36	13,22	14,46	17,07	19,72	22,37	24,95	26,19	28,01
**13,5**	11,49	13,31	14,54	17,11	19,75	22,39	24,97	26,21	28,04
**14**	11,57	13,36	14,57	17,12	19,74	22,38	24,96	26,20	28,04
**14,5**	11,61	13,38	14,58	17,10	19,71	22,35	24,94	26,18	28,04
**15**	11,63	13,37	14,56	17,07	19,68	22,32	24,92	26,17	28,04
**15,5**	11,62	13,35	14,53	17,04	19,64	22,29	24,91	26,18	28,07
**16**	11,60	13,32	14,50	17,00	19,62	22,28	24,93	26,21	28,12
**16,5**	11,57	13,29	14,46	16,97	19,60	22,29	24,97	26,27	28,21
**17**	11,54	13,25	14,43	16,95	19,60	22,32	25,04	26,36	28,34

Tab. 4: Push-up Percentiles for the representative sample of German boys and girls aged 4-17y (MoMo Wave 1)

Boys Push-ups [Number in 40 sec]

Age (years)	5%	10%	15%	30%	50%	70%	85%	90%	95%
**6**	4,86	5,72	6,32	7,63	9,05	10,54	12,06	12,81	13,94
**6,5**	5,11	6,02	6,65	8,03	9,51	11,05	12,62	13,39	14,55
**7**	5,37	6,33	6,99	8,43	9,97	11,56	13,17	13,96	15,15
**7,5**	5,63	6,63	7,33	8,83	10,42	12,06	13,71	14,52	15,74
**8**	5,88	6,93	7,65	9,21	10,85	12,54	14,24	15,06	16,30
**8,5**	6,11	7,20	7,95	9,56	11,25	12,99	14,72	15,57	16,83
**9**	6,30	7,43	8,21	9,87	11,61	13,38	15,15	16,00	17,28
**9,5**	6,45	7,62	8,42	10,12	11,90	13,71	15,50	16,36	17,65

**10**	6,57	7,78	8,60	10,34	12,15	13,98	15,78	16,65	17,94
**10,5**	6,67	7,91	8,75	10,53	12,36	14,20	16,01	16,88	18,17
**11**	6,77	8,04	8,90	10,70	12,55	14,40	16,21	17,07	18,36
**11,5**	6,89	8,18	9,06	10,88	12,73	14,58	16,39	17,25	18,53
**12**	7,04	8,36	9,24	11,07	12,93	14,78	16,57	17,43	18,69
**12,5**	7,24	8,56	9,45	11,28	13,14	14,98	16,77	17,62	18,88
**13**	7,47	8,80	9,68	11,52	13,37	15,22	17,00	17,85	19,10
**13,5**	7,73	9,06	9,94	11,78	13,64	15,48	17,26	18,11	19,36
**14**	8,00	9,33	10,22	12,06	13,92	15,77	17,55	18,40	19,65
**14,5**	8,28	9,62	10,51	12,36	14,22	16,07	17,85	18,70	19,96
**15**	8,56	9,90	10,80	12,66	14,53	16,38	18,17	19,02	20,28
**15,5**	8,84	10,19	11,09	12,96	14,84	16,70	18,49	19,34	20,60
**16**	9,12	10,48	11,39	13,26	15,14	17,01	18,81	19,66	20,92
**16,5**	9,41	10,77	11,67	13,55	15,44	17,31	19,11	19,96	21,23
**17**	9,69	11,05	11,96	13,83	15,72	17,59	19,39	20,25	21,52

Girls Push-ups [Number in 40 sec]

**Age (years)**	**5%**	**10%**	**15%**	**30%**	**50%**	**70%**	**85%**	**90%**	**95%**
**6**	5,59	6,48	7,10	8,39	9,74	11,12	12,48	13,14	14,12
**6,5**	5,81	6,74	7,37	8,72	10,13	11,56	12,99	13,68	14,70
**7**	6,03	6,99	7,64	9,04	10,50	12,00	13,48	14,20	15,27
**7,5**	6,24	7,22	7,90	9,34	10,86	12,41	13,95	14,70	15,82
**8**	6,44	7,45	8,14	9,63	11,19	12,80	14,40	15,17	16,34
**8,5**	6,64	7,67	8,38	9,90	11,50	13,16	14,81	15,61	16,82
**9**	6,81	7,86	8,59	10,14	11,78	13,48	15,18	16,01	17,25
**9,5**	6,96	8,02	8,76	10,34	12,02	13,75	15,48	16,33	17,60
**10**	7,06	8,14	8,88	10,49	12,19	13,94	15,71	16,56	17,86
**10,5**	7,12	8,21	8,96	10,59	12,30	14,07	15,84	16,70	18,00
**11**	7,14	8,25	9,01	10,64	12,36	14,13	15,90	16,76	18,05
**11,5**	7,15	8,27	9,04	10,68	12,40	14,16	15,91	16,76	18,04
**12**	7,15	8,28	9,05	10,70	12,41	14,16	15,89	16,73	17,98
**12,5**	7,14	8,28	9,06	10,71	12,41	14,14	15,85	16,67	17,89
**13**	7,13	8,28	9,06	10,70	12,40	14,10	15,78	16,58	17,78
**13,5**	7,12	8,28	9,06	10,69	12,37	14,05	15,70	16,49	17,66
**14**	7,11	8,28	9,06	10,69	12,35	14,01	15,63	16,40	17,54
**14,5**	7,11	8,28	9,06	10,68	12,33	13,97	15,57	16,32	17,45
**15**	7,12	8,29	9,07	10,69	12,33	13,96	15,53	16,28	17,38
**15,5**	7,15	8,32	9,11	10,73	12,36	13,97	15,53	16,27	17,36
**16**	7,20	8,38	9,17	10,79	12,42	14,02	15,57	16,31	17,39
**16,5**	7,28	8,46	9,25	10,88	12,51	14,12	15,67	16,41	17,49
**17**	7,37	8,56	9,36	10,99	12,63	14,25	15,81	16,55	17,64

Tab. 5: Standing long jump Percentiles for the representative sample of German boys and girls aged 4-17y (MoMo Wave 1)

Boys Standing long jump [cm]

Age (years)	5%	10%	15%	30%	50%	70%	85%	90%	95%
**4**	55,57	62,07	66,31	74,83	83,18	91,21	98,79	102,34	107,51
**4,5**	58,63	65,35	69,73	78,53	87,13	95,39	103,18	106,82	112,13
**5**	63,05	70,08	74,65	83,81	92,75	101,32	109,39	113,15	118,63
**5,5**	67,54	74,86	79,61	89,12	98,37	107,22	115,53	119,41	125,05
**6**	72,07	79,66	84,57	94,39	103,93	113,03	121,57	125,55	131,33
**6,5**	76,62	84,44	89,49	99,59	109,37	118,71	127,45	131,52	137,43
**7**	81,17	89,17	94,34	104,66	114,67	124,20	133,13	137,29	143,32
**7,5**	85,70	93,83	99,09	109,59	119,78	129,50	138,59	142,83	148,98
**8**	90,12	98,35	103,68	114,33	124,68	134,55	143,80	148,10	154,35
**8,5**	94,32	102,64	108,04	118,83	129,31	139,32	148,69	153,06	159,40
**9**	98,24	106,67	112,13	123,06	133,67	143,79	153,27	157,69	164,10
**9,5**	101,89	110,44	115,98	127,05	137,78	148,02	157,60	162,05	168,52
**10**	105,29	114,00	119,63	130,87	141,74	152,08	161,75	166,24	172,75
**10,5**	108,46	117,38	123,13	134,57	145,60	156,06	165,81	170,33	176,88
**11**	111,46	120,64	126,53	138,21	149,42	160,02	169,86	174,42	181,01
**11,5**	114,37	123,85	129,92	141,89	153,32	164,07	174,03	178,63	185,27
**12**	117,28	127,12	133,39	145,68	157,37	168,32	178,41	183,07	189,78
**12,5**	120,28	130,52	137,00	149,66	161,64	172,80	183,07	187,79	194,58
**13**	123,47	134,09	140,80	153,84	166,11	177,52	187,97	192,77	199,67
**13,5**	126,92	137,89	144,80	158,19	170,76	182,41	193,07	197,95	204,96
**14**	130,71	141,93	148,99	162,68	175,50	187,38	198,25	203,22	210,37
**14,5**	134,78	146,14	153,30	167,19	180,23	192,32	203,38	208,45	215,73
**15**	138,95	150,37	157,58	171,61	184,81	197,08	208,32	213,48	220,89
**15,5**	143,03	154,46	161,69	175,81	189,13	201,54	212,93	218,17	225,69
**16**	146,92	158,32	165,56	179,72	193,13	205,64	217,16	222,45	230,07
**16,5**	150,53	161,89	169,12	183,30	196,76	209,36	220,96	226,30	233,99
**17**	153,79	165,11	172,33	186,51	200,01	212,66	224,33	229,70	237,45

Girls Standing long jump [cm]

Age (years)	5%	10%	15%	30%	50%	70%	85%	90%	95%
**4**	58,98	63,71	66,81	73,09	79,27	85,23	90,87	93,52	97,38
**4,5**	62,40	67,46	70,76	77,41	83,93	90,20	96,12	98,88	102,91
**5**	67,10	72,60	76,17	83,35	90,34	97,02	103,30	106,23	110,48
**5,5**	71,73	77,68	81,53	89,21	96,65	103,74	110,37	113,45	117,92
**6**	76,25	82,63	86,74	94,91	102,79	110,27	117,23	120,46	125,14
**6,5**	80,60	87,38	91,73	100,36	108,65	116,49	123,78	127,15	132,04
**7**	84,72	91,84	96,41	105,46	114,13	122,31	129,91	133,43	138,51
**7,5**	88,57	95,97	100,72	110,12	119,13	127,64	135,54	139,19	144,48
**8**	92,16	99,76	104,65	114,34	123,66	132,46	140,64	144,43	149,92

**8,5**	95,51	103,24	108,23	118,14	127,72	136,79	145,26	149,19	154,87
**9**	98,62	106,41	111,45	121,54	131,34	140,68	149,41	153,48	159,38
**9,5**	101,45	109,24	114,33	124,54	134,53	144,11	153,12	157,33	163,45
**10**	103,98	111,74	116,84	127,15	137,31	147,12	156,41	160,76	167,11
**10,5**	106,19	113,91	119,00	129,39	139,71	149,74	159,30	163,80	170,38
**11**	108,11	115,77	120,86	131,30	141,76	152,01	161,84	166,49	173,32
**11,5**	109,78	117,37	122,45	132,94	143,54	154,00	164,10	168,90	175,97
**12**	111,21	118,75	123,82	134,35	145,07	155,74	166,09	171,03	178,34
**12,5**	112,42	119,91	124,98	135,55	146,40	157,25	167,85	172,92	180,45
**13**	113,41	120,89	125,95	136,58	147,54	158,56	169,39	174,59	182,33
**13,5**	114,21	121,68	126,75	137,44	148,51	159,70	170,73	176,05	183,98
**14**	114,83	122,30	127,39	138,14	149,32	160,66	171,88	177,30	185,40
**14,5**	115,30	122,79	127,89	138,71	149,99	161,47	172,86	178,38	186,62
**15**	115,67	123,18	128,31	139,19	150,57	162,17	173,70	179,29	187,66
**15,5**	115,98	123,51	128,66	139,61	151,07	162,77	174,43	180,09	188,57
**16**	116,30	123,84	129,01	140,01	151,54	163,33	175,10	180,81	189,39
**16,5**	116,65	124,21	129,39	140,42	152,01	163,88	175,73	181,50	190,15
**17**	117,03	124,60	129,80	140,86	152,49	164,42	176,34	182,14	190,85

Tab.6: Jumping side-ways Percentiles for the representative sample of German boys and girls aged 4-17y (MoMo Wave 1)

Boys Jumping side-ways [Number in 15 sec]

**Age (years)**	**5%**	**10%**	**15%**	**30%**	**50%**	**70%**	**85%**	**90%**	**95%**
**4**	5,06	5,89	6,48	7,81	9,28	10,88	12,56	13,40	14,70
**4,5**	5,78	6,70	7,36	8,83	10,46	12,22	14,07	14,99	16,42
**5**	6,82	7,87	8,63	10,29	12,14	14,12	16,19	17,23	18,82
**5,5**	7,91	9,10	9,94	11,80	13,85	16,04	18,32	19,46	21,20
**6**	9,05	10,36	11,30	13,35	15,59	17,98	20,45	21,68	23,56
**6,5**	10,23	11,68	12,70	14,93	17,36	19,93	22,58	23,89	25,89
**7**	11,45	13,02	14,12	16,53	19,13	21,87	24,67	26,05	28,15
**7,5**	12,68	14,38	15,57	18,14	20,90	23,78	26,71	28,15	30,33
**8**	13,92	15,74	17,00	19,72	22,62	25,63	28,67	30,15	32,40
**8,5**	15,13	17,07	18,40	21,26	24,28	27,39	30,50	32,02	34,30
**9**	16,31	18,35	19,75	22,73	25,85	29,03	32,20	33,74	36,04
**9,5**	17,43	19,58	21,04	24,12	27,32	30,57	33,77	35,31	37,61
**10**	18,52	20,76	22,28	25,45	28,72	32,00	35,22	36,76	39,06
**10,5**	19,57	21,90	23,46	26,72	30,04	33,35	36,57	38,11	40,38
**11**	20,57	22,98	24,60	27,93	31,29	34,61	37,82	39,34	41,59
**11,5**	21,51	24,01	25,67	29,06	32,46	35,79	38,98	40,49	42,70
**12**	22,39	24,98	26,68	30,14	33,56	36,89	40,05	41,54	43,72
**12,5**	23,22	25,88	27,63	31,14	34,59	37,91	41,05	42,52	44,67

**13**	24,00	26,74	28,52	32,07	35,54	38,85	41,97	43,42	45,54
**13,5**	24,76	27,55	29,35	32,94	36,41	39,71	42,80	44,23	46,32
**14**	25,51	28,33	30,14	33,73	37,19	40,47	43,53	44,95	47,01
**14,5**	26,24	29,06	30,86	34,44	37,88	41,13	44,16	45,57	47,60
**15**	26,94	29,74	31,53	35,08	38,49	41,72	44,71	46,10	48,12
**15,5**	27,58	30,36	32,14	35,66	39,04	42,24	45,20	46,58	48,57
**16**	28,16	30,92	32,69	36,19	39,54	42,71	45,65	47,02	48,99
**16,5**	28,68	31,43	33,19	36,67	40,00	43,15	46,07	47,43	49,39
**17**	29,14	31,88	33,63	37,10	40,42	43,55	46,46	47,81	49,76

Girls Jumping side-ways [Number in 15 sec]

**Age (years)**	**5%**	**10%**	**15%**	**30%**	**50%**	**70%**	**85%**	**90%**	**95%**
**4**	5,37	6,32	6,99	8,44	10,01	11,65	13,31	14,13	15,37
**4,5**	6,18	7,28	8,04	9,69	11,45	13,27	15,11	16,01	17,37
**5**	7,34	8,62	9,51	11,41	13,42	15,48	17,54	18,54	20,04
**5,5**	8,53	10,00	11,01	13,15	15,38	17,65	19,91	20,99	22,62
**6**	9,78	11,43	12,54	14,90	17,34	19,80	22,22	23,39	25,13
**6,5**	11,08	12,89	14,10	16,65	19,27	21,90	24,47	25,71	27,53
**7**	12,42	14,36	15,66	18,38	21,15	23,92	26,61	27,90	29,80
**7,5**	13,76	15,82	17,20	20,05	22,95	25,82	28,60	29,93	31,88
**8**	15,10	17,25	18,69	21,65	24,64	27,59	30,43	31,78	33,77
**8,5**	16,42	18,65	20,13	23,16	26,21	29,20	32,08	33,44	35,45
**9**	17,69	19,97	21,48	24,56	27,64	30,66	33,55	34,91	36,92
**9,5**	18,89	21,20	22,73	25,84	28,93	31,95	34,83	36,19	38,19
**10**	20,00	22,33	23,86	26,98	30,08	33,08	35,95	37,30	39,28
**10,5**	21,01	23,35	24,88	28,00	31,09	34,08	36,92	38,26	40,21
**11**	21,91	24,25	25,79	28,90	31,97	34,94	37,76	39,08	41,02
**11,5**	22,72	25,06	26,59	29,70	32,75	35,71	38,50	39,81	41,73
**12**	23,42	25,77	27,30	30,40	33,45	36,39	39,17	40,47	42,38
**12,5**	24,04	26,39	27,92	31,02	34,06	37,00	39,78	41,08	42,98
**13**	24,57	26,91	28,45	31,56	34,61	37,56	40,34	41,65	43,56
**13,5**	25,01	27,36	28,90	32,02	35,09	38,05	40,86	42,18	44,10
**14**	25,37	27,73	29,28	32,41	35,50	38,50	41,34	42,67	44,62
**14,5**	25,68	28,04	29,59	32,75	35,87	38,90	41,78	43,14	45,12
**15**	25,95	28,31	29,87	33,04	36,20	39,27	42,20	43,58	45,61
**15,5**	26,20	28,56	30,11	33,31	36,50	39,62	42,61	44,02	46,09
**16**	26,44	28,79	30,35	33,56	36,78	39,96	43,01	44,45	46,57
**16,5**	26,67	29,01	30,57	33,79	37,06	40,28	43,39	44,87	47,05
**17**	26,90	29,22	30,78	34,02	37,31	40,58	43,76	45,27	47,51

Tab.7: Static stand Percentiles for the representative sample of German boys and girls aged 4-17y (MoMo Wave 1)

Boys Static stand [Number of ground contacts in 60 sec]

Age (years)	5%	10%	15%	30%	50%	70%	85%	90%	95%
**4**	29,70	27,21	25,53	22,01	18,38	14,73	11,14	9,41	6,82
**4,5**	29,43	26,84	25,09	21,44	17,71	13,99	10,37	8,64	6,09
**5**	28,86	26,07	24,20	20,34	16,46	12,67	9,06	7,38	4,96
**5,5**	28,19	25,21	23,24	19,21	15,23	11,42	7,91	6,31	4,08
**6**	27,42	24,27	22,20	18,03	14,00	10,25	6,90	5,42	3,41
**6,5**	26,56	23,25	21,09	16,83	12,80	9,16	6,02	4,68	2,91
**7**	25,60	22,14	19,92	15,60	11,63	8,15	5,26	4,06	2,52
**7,5**	24,55	20,96	18,70	14,37	10,50	7,23	4,60	3,54	2,22
**8**	23,41	19,73	17,43	13,14	9,43	6,38	4,02	3,10	1,97
**8,5**	22,25	18,48	16,18	11,96	8,42	5,62	3,52	2,73	1,77
**9**	21,10	17,26	14,96	10,85	7,51	4,95	3,10	2,41	1,59
**9,5**	19,98	16,09	13,81	9,81	6,68	4,36	2,73	2,13	1,43
**10**	18,92	14,98	12,72	8,87	5,94	3,84	2,41	1,90	1,30
**10,5**	17,94	13,97	11,73	8,02	5,30	3,40	2,14	1,69	1,18
**11**	17,12	13,10	10,88	7,30	4,76	3,04	1,92	1,53	1,08
**11,5**	16,53	12,42	10,20	6,72	4,33	2,75	1,75	1,40	1,00
**12**	16,19	11,94	9,71	6,28	4,00	2,53	1,62	1,30	0,94
**12,5**	16,09	11,65	9,38	5,97	3,76	2,38	1,52	1,23	0,90
**13**	16,19	11,52	9,19	5,76	3,60	2,27	1,46	1,18	0,87
**13,5**	16,42	11,51	9,10	5,63	3,50	2,20	1,42	1,16	0,86
**14**	16,67	11,53	9,05	5,54	3,42	2,15	1,39	1,14	0,85
**14,5**	16,83	11,52	8,99	5,45	3,35	2,11	1,37	1,12	0,84
**15**	16,86	11,45	8,89	5,36	3,29	2,06	1,34	1,10	0,82
**15,5**	16,76	11,31	8,76	5,26	3,21	2,02	1,31	1,08	0,81
**16**	16,54	11,11	8,59	5,14	3,14	1,97	1,28	1,05	0,79
**16,5**	16,25	10,89	8,40	5,02	3,06	1,92	1,25	1,03	0,77
**17**	15,95	10,68	8,23	4,92	3,00	1,88	1,23	1,01	0,76

Girls Static stand [Number of ground contacts in 60 sec]

Age (years)	5%	10%	15%	30%	50%	70%	85%	90%	95%
**4**	27,58	24,40	22,31	18,08	13,97	10,10	6,63	5,09	2,99
**4,5**	26,86	23,64	21,52	17,28	13,21	9,44	6,12	4,68	2,74
**5**	25,81	22,52	20,38	16,15	12,16	8,56	5,47	4,16	2,45
**5,5**	24,68	21,34	19,19	14,99	11,12	7,71	4,87	3,69	2,19
**6**	23,48	20,09	17,95	13,81	10,09	6,90	4,31	3,27	1,97
**6,5**	22,21	18,80	16,67	12,63	9,08	6,13	3,81	2,89	1,77
**7**	20,90	17,49	15,39	11,47	8,12	5,41	3,35	2,55	1,59
**7,5**	19,60	16,19	14,12	10,35	7,22	4,75	2,94	2,25	1,43

**8**	18,33	14,94	12,92	9,30	6,39	4,17	2,57	1,98	1,29
**8,5**	17,15	13,77	11,79	8,34	5,64	3,65	2,26	1,75	1,16
**9**	16,09	12,73	10,79	7,49	4,99	3,21	1,99	1,55	1,05
**9,5**	15,23	11,84	9,93	6,77	4,45	2,84	1,77	1,39	0,96
**10**	14,60	11,15	9,25	6,18	4,01	2,55	1,60	1,27	0,89
**10,5**	14,20	10,64	8,73	5,72	3,67	2,32	1,47	1,17	0,83
**11**	14,00	10,29	8,35	5,38	3,41	2,15	1,37	1,10	0,79
**11,5**	13,96	10,06	8,08	5,11	3,21	2,02	1,29	1,05	0,76
**12**	14,03	9,93	7,89	4,92	3,07	1,93	1,24	1,01	0,74
**12,5**	14,17	9,88	7,79	4,80	2,97	1,87	1,21	0,98	0,73
**13**	14,36	9,88	7,73	4,72	2,91	1,83	1,19	0,97	0,72
**13,5**	14,53	9,90	7,71	4,67	2,87	1,81	1,18	0,96	0,72
**14**	14,66	9,93	7,71	4,65	2,85	1,80	1,17	0,96	0,73
**14,5**	14,73	9,95	7,71	4,65	2,85	1,80	1,18	0,97	0,73
**15**	14,78	9,98	7,74	4,67	2,87	1,82	1,19	0,98	0,74
**15,5**	14,84	10,05	7,80	4,72	2,91	1,84	1,21	1,00	0,75
**16**	14,86	10,10	7,86	4,78	2,95	1,88	1,23	1,02	0,77
**16,5**	14,78	10,10	7,89	4,82	2,99	1,91	1,26	1,04	0,78
**17**	14,59	10,03	7,86	4,82	3,01	1,92	1,27	1,05	0,80

Tab.8: Balancing backwards Percentiles for the representative sample of German boys and girls aged 4-17y (MoMo Wave 1)

Boys Balancing backwards [Sum of steps of 6 trials]

**Age (years)**	**5%**	**10%**	**15%**	**30%**	**50%**	**70%**	**85%**	**90%**	**95%**
**4**	3,25	4,55	5,54	7,96	10,90	14,11	17,78	19,63	23,22
**4,5**	3,77	5,26	6,39	9,11	12,36	15,87	19,82	21,79	25,58
**5**	4,55	6,32	7,66	10,78	14,45	18,32	22,59	24,71	28,73
**5,5**	5,40	7,46	8,99	12,50	16,53	20,70	25,24	27,47	31,65
**6**	6,33	8,68	10,39	14,25	18,59	23,01	27,75	30,05	34,33
**6,5**	7,33	9,96	11,84	16,03	20,64	25,25	30,12	32,45	36,78
**7**	8,39	11,30	13,34	17,82	22,64	27,39	32,33	34,68	39,00
**7,5**	9,52	12,68	14,88	19,59	24,57	29,41	34,37	36,71	40,98
**8**	10,67	14,08	16,41	21,32	26,42	31,28	36,22	38,53	42,70
**8,5**	11,83	15,47	17,91	22,97	28,13	32,98	37,84	40,09	44,14
**9**	12,98	16,81	19,34	24,51	29,67	34,46	39,20	41,38	45,28
**9,5**	14,09	18,09	20,69	25,91	31,03	35,72	40,31	42,40	46,13
**10**	15,16	19,30	21,94	27,16	32,21	36,77	41,19	43,19	46,73
**10,5**	16,18	20,42	23,08	28,28	33,23	37,65	41,88	43,79	47,15
**11**	17,14	21,45	24,13	29,28	34,11	38,38	42,43	44,26	47,45
**11,5**	18,02	22,39	25,07	30,16	34,87	38,99	42,88	44,62	47,65

**12**	18,82	23,24	25,90	30,92	35,52	39,51	43,25	44,91	47,80
**12,5**	19,52	23,98	26,64	31,60	36,09	39,96	43,56	45,16	47,93
**13**	20,15	24,64	27,29	32,19	36,59	40,35	43,83	45,37	48,03
**13,5**	20,71	25,22	27,86	32,70	37,02	40,68	44,07	45,56	48,13
**14**	21,25	25,76	28,39	33,17	37,40	40,99	44,28	45,73	48,22
**14,5**	21,77	26,27	28,87	33,60	37,76	41,26	44,48	45,89	48,32
**15**	22,27	26,75	29,33	33,99	38,09	41,53	44,68	46,06	48,43
**15,5**	22,74	27,19	29,75	34,35	38,39	41,78	44,87	46,22	48,54
**16**	23,16	27,59	30,12	34,68	38,67	42,00	45,04	46,37	48,65
**16,5**	23,53	27,93	30,44	34,96	38,90	42,19	45,18	46,49	48,74
**17**	23,86	28,22	30,71	35,18	39,07	42,32	45,27	46,56	48,78

Girls Balancing backwards [Sum of steps of 6 trials]

**Age (years)**	**5%**	**10%**	**15%**	**30%**	**50%**	**70%**	**85%**	**90%**	**95%**
**4**	6,29	7,63	8,63	10,98	13,79	17,03	20,63	22,52	25,52
**4,5**	7,02	8,56	9,71	12,36	15,46	18,96	22,76	24,73	27,80
**5**	8,00	9,86	11,21	14,28	17,75	21,56	25,59	27,63	30,78
**5,5**	8,98	11,16	12,72	16,19	20,01	24,09	28,30	30,39	33,57
**6**	9,95	12,48	14,25	18,10	22,23	26,53	30,86	32,99	36,19
**6,5**	10,95	13,82	15,79	19,99	24,38	28,85	33,28	35,42	38,62
**7**	11,95	15,16	17,31	21,82	26,42	31,02	35,50	37,65	40,82
**7,5**	12,97	16,49	18,81	23,57	28,33	33,01	37,51	39,64	42,78
**8**	14,01	17,80	20,26	25,22	30,09	34,81	39,29	41,40	44,48
**8,5**	15,07	19,09	21,66	26,76	31,69	36,41	40,84	42,91	45,93
**9**	16,13	20,33	22,98	28,17	33,12	37,80	42,16	44,18	47,12
**9,5**	17,19	21,52	24,22	29,44	34,37	38,98	43,25	45,22	48,07
**10**	18,23	22,64	25,36	30,58	35,45	39,97	44,12	46,03	48,79
**10,5**	19,23	23,68	26,40	31,57	36,36	40,77	44,80	46,65	49,31
**11**	20,17	24,63	27,33	32,43	37,11	41,40	45,30	47,09	49,65
**11,5**	21,05	25,48	28,15	33,16	37,73	41,90	45,66	47,38	49,84
**12**	21,84	26,25	28,88	33,79	38,24	42,28	45,91	47,56	49,93
**12,5**	22,55	26,92	29,51	34,33	38,66	42,57	46,07	47,66	49,93
**13**	23,16	27,50	30,06	34,78	39,00	42,78	46,16	47,69	49,86
**13,5**	23,67	28,01	30,54	35,17	39,28	42,94	46,20	47,67	49,76
**14**	24,12	28,45	30,96	35,51	39,51	43,07	46,21	47,63	49,64
**14,5**	24,50	28,84	31,33	35,81	39,73	43,18	46,22	47,59	49,52
**15**	24,83	29,20	31,67	36,10	39,93	43,29	46,24	47,56	49,42
**15,5**	25,13	29,53	32,00	36,37	40,12	43,40	46,26	47,54	49,34
**16**	25,42	29,85	32,31	36,63	40,31	43,51	46,29	47,53	49,27
**16,5**	25,71	30,17	32,61	36,89	40,51	43,63	46,34	47,55	49,24
**17**	26,01	30,49	32,93	37,15	40,71	43,77	46,41	47,58	49,22

Tab.9: Stand and reach Percentiles for the representative sample of German boys and girls aged 4-17y (MoMo Wave 1)

Boys Stand Reach [Distance between fingertips and zero level in cm]

Age (years)	5%	10%	15%	30%	50%	70%	85%	90%	95%
**4**	-7,53	-5,56	-4,29	-1,74	0,71	3,06	5,24	6,26	7,73
**4,5**	-7,74	-5,72	-4,42	-1,81	0,71	3,11	5,35	6,40	7,91
**5**	-8,04	-5,95	-4,60	-1,91	0,70	3,18	5,51	6,59	8,16
**5,5**	-8,34	-6,19	-4,80	-2,02	0,68	3,25	5,65	6,77	8,40
**6**	-8,66	-6,45	-5,01	-2,15	0,64	3,29	5,78	6,94	8,62
**6,5**	-9,00	-6,72	-5,25	-2,30	0,57	3,31	5,89	7,09	8,83
**7**	-9,37	-7,03	-5,51	-2,48	0,48	3,31	5,97	7,21	9,01
**7,5**	-9,76	-7,36	-5,81	-2,69	0,35	3,26	6,00	7,28	9,15
**8**	-10,17	-7,73	-6,14	-2,95	0,17	3,17	5,99	7,31	9,23
**8,5**	-10,62	-8,13	-6,51	-3,25	-0,05	3,03	5,93	7,29	9,27
**9**	-11,09	-8,57	-6,92	-3,59	-0,33	2,83	5,81	7,21	9,25
**9,5**	-11,60	-9,04	-7,37	-3,99	-0,65	2,58	5,64	7,08	9,18
**10**	-12,12	-9,54	-7,85	-4,41	-1,01	2,29	5,44	6,91	9,08
**10,5**	-12,66	-10,06	-8,35	-4,86	-1,40	1,98	5,21	6,73	8,96
**11**	-13,19	-10,58	-8,85	-5,32	-1,80	1,65	4,96	6,52	8,82
**11,5**	-13,71	-11,09	-9,35	-5,78	-2,19	1,34	4,73	6,34	8,71
**12**	-14,19	-11,56	-9,81	-6,19	-2,54	1,07	4,55	6,20	8,65
**12,5**	-14,63	-11,99	-10,22	-6,55	-2,83	0,86	4,44	6,14	8,66
**13**	-15,01	-12,35	-10,56	-6,84	-3,05	0,73	4,40	6,15	8,75
**13,5**	-15,34	-12,65	-10,84	-7,06	-3,19	0,67	4,44	6,25	8,92
**14**	-15,61	-12,89	-11,05	-7,21	-3,26	0,70	4,57	6,43	9,18
**14,5**	-15,82	-13,06	-11,19	-7,27	-3,23	0,82	4,80	6,71	9,54
**15**	-15,99	-13,17	-11,27	-7,26	-3,12	1,04	5,12	7,08	9,99
**15,5**	-16,11	-13,23	-11,28	-7,18	-2,94	1,33	5,52	7,53	10,53
**16**	-16,20	-13,25	-11,25	-7,04	-2,70	1,68	5,99	8,06	11,13
**16,5**	-16,27	-13,25	-11,19	-6,87	-2,41	2,08	6,50	8,62	11,77
**17**	-16,34	-13,24	-11,13	-6,69	-2,12	2,48	7,00	9,17	12,39

Girls Stand Reach [Distance between fingertips and zero level in cm]

Age (years)	5%	10%	15%	30%	50%	70%	85%	90%	95%
**4**	-2,33	-1,02	-0,15	1,58	3,28	4,92	6,45	7,17	8,21
**4,5**	-2,65	-1,23	-0,31	1,55	3,37	5,11	6,75	7,52	8,63
**5**	-3,08	-1,53	-0,52	1,51	3,49	5,38	7,16	7,99	9,20
**5,5**	-3,52	-1,84	-0,75	1,45	3,60	5,65	7,57	8,47	9,77
**6**	-4,00	-2,18	-1,00	1,37	3,69	5,89	7,97	8,93	10,33
**6,5**	-4,53	-2,57	-1,30	1,25	3,74	6,11	8,33	9,37	10,87
**7**	-5,08	-2,98	-1,62	1,10	3,75	6,29	8,67	9,78	11,39
**7,5**	-5,64	-3,41	-1,97	0,92	3,74	6,44	8,98	10,16	11,88
**8**	-6,19	-3,85	-2,32	0,74	3,73	6,59	9,28	10,54	12,37

**8,5**	-6,73	-4,27	-2,67	0,55	3,70	6,73	9,58	10,92	12,86
**9**	-7,25	-4,68	-3,01	0,37	3,68	6,87	9,88	11,29	13,35
**9,5**	-7,75	-5,09	-3,34	0,18	3,64	6,99	10,15	11,64	13,80
**10**	-8,23	-5,47	-3,66	-0,01	3,60	7,08	10,39	11,95	14,22
**10,5**	-8,65	-5,81	-3,95	-0,18	3,56	7,18	10,63	12,25	14,62
**11**	-9,00	-6,10	-4,19	-0,31	3,54	7,29	10,87	12,55	15,02
**11,5**	-9,28	-6,33	-4,38	-0,41	3,55	7,42	11,12	12,86	15,43
**12**	-9,49	-6,50	-4,52	-0,46	3,59	7,57	11,38	13,18	15,83
**12,5**	-9,65	-6,62	-4,61	-0,49	3,65	7,72	11,63	13,49	16,22
**13**	-9,76	-6,71	-4,68	-0,49	3,72	7,88	11,88	13,78	16,58
**13,5**	-9,84	-6,76	-4,71	-0,47	3,81	8,04	12,12	14,06	16,92
**14**	-9,90	-6,79	-4,72	-0,43	3,91	8,20	12,35	14,32	17,23
**14,5**	-9,95	-6,81	-4,72	-0,38	4,02	8,36	12,57	14,57	17,52
**15**	-10,01	-6,84	-4,72	-0,33	4,11	8,51	12,76	14,78	17,77
**15,5**	-10,10	-6,88	-4,74	-0,30	4,19	8,62	12,91	14,95	17,95
**16**	-10,22	-6,96	-4,78	-0,29	4,23	8,70	13,01	15,06	18,07
**16,5**	-10,37	-7,05	-4,85	-0,31	4,26	8,75	13,07	15,12	18,14
**17**	-10,56	-7,17	-4,93	-0,34	4,27	8,78	13,11	15,16	18,18
